# Commentary: Editorial: The convergence of AI, LLMs, and industry 4.0: enhancing BCI, HMI, and neuroscience research

**DOI:** 10.3389/fncom.2026.1810869

**Published:** 2026-04-13

**Authors:** Alessandro Rossi

**Affiliations:** 1 Department of Neurosciences, University of Siena, Siena, Italy; 2 Rehabilitation Research Foundation Gianfranco Salvini, ETS, Montevarchi, Arezzo, Italy

**Keywords:** EEG, BCI, LLM, neuroergonomics, hard problem of consciousness, research methodology, AI ethics

## 1 Introduction

The ambition of Asgher (2025)'s editorial is not in question. Framing the intersection of Brain–Computer Interfaces (BCI), Large Language Models (LLMs), and Industry 4.0 deployment infrastructure as a unified engineering and scientific challenge is a legitimate and timely intellectual move. The four-layer model proposed, interaction, measurement, inference, and deployment, offers a useful schematic for a community whose sub-fields often proceed in mutual ignorance.

Yet an editorial that positions itself as defining a research agenda for neuroadaptive human-AI systems must be held to the standards it implicitly invokes. When a text asserts that it is grounded in a “concrete engineering and scientific shift” and that four articles constitute “a credible cross-section” of the field, it makes empirical and epistemological claims that are open to scrutiny. The present commentary argues that these claims do not withstand examination, and that the editorial's principal weaknesses are not incidental but structural.

The critique proceeds in seven sections, moving from conceptual to methodological to ethical concerns, before concluding with what we consider the deepest problem: the unexamined inferential gap between the electrical signals measured by EEG and the mental states whose properties are claimed to be known.

## 2 “Convergence” as rhetorical rather than empirical category

In his editorial, Asgher (2025) frames convergence through three interlocking pillars: first, AI models that infer and adapt to human cognitive and affective states via neuroadaptive control; second, LLM-centric interaction layers serving as cognitive interfaces between humans and complex systems; and third, Industry 4.0 deployment substrates integrating these capabilities into cyber-physical systems with latency, reliability, and governance constraints. He further proposes a closed-loop socio-technical control model with four layers: an interaction layer (LLM/HMI), a measurement layer (BCI/neuroergonomics), an inference and policy layer (AI/RL), and a deployment layer (Industry 4.0/CPS).

The editorial opens by distinguishing its use of “convergence” from mere interdisciplinary work, describing it as “a concrete engineering and scientific shift toward closed-loop human–AI systems.” This distinction is stated but never demonstrated. For convergence to be a scientific claim rather than a programmatic aspiration, one would need to show that previously separate fields are developing shared protocols, common measurement standards, or a unified technical vocabulary. None of this is established by the four articles cited, which remain disciplinarily distinct contributions: philosophical AI alignment theory, a small-N neuroergonomics pilot, a clinical NLP classification pipeline, and a computational linguistics representational analysis.

The admission in the editorial's conclusion that “the field is still largely assembling components” is internally inconsistent with the opening claim of ongoing convergence. If the components are not yet assembled, the convergence has not occurred, it is at best a normative goal. This conflation of descriptive and prescriptive registers is a recurring feature of what has been called “promissory science” (Nordmann, 2007): the practice of treating hoped-for futures as accomplished presents in order to generate research momentum and funding legitimacy.

A more defensible framing would have been explicit about this prescriptive character: not “convergence is happening” but “convergence is needed, here is what it would require, and here are four partial steps in that direction.” The difference is not semantic; it determines the evidential standards to which the work can be held.

## 3 An empirical base insufficient for agenda-setting

Asgher (2025)'s editorial synthesizes four published articles as instantiations of the convergence agenda. Edwards provides a conceptual and architectural argument for safe AI through perspective-taking and value-grounded behavior, proposing a neuro-symbolic alignment approach. Jiang et al. present an EEG-based investigation of how LLM assistance modulates cognition during problem-solving, including a pilot study with 12 participants comparing GPT-4-assisted and solo reasoning conditions. Li et al. contribute a text classification pipeline (Bert-BiLSTM-Att_dropout) for triaging adverse events from da Vinci surgical robot MAUDE reports (2013–2023), achieving an average F1 of approximately 90.15. Ramezani et al. analyze whether an LSTM language model develops internal representations that separate argument structure constructions, using controlled GPT-4-generated stimuli and representational analysis techniques. Asgher (2025) characterizes these four contributions as forming a “coherent research mosaic” that provides a “credible cross-section” of what convergence currently looks like in the literature.

The editorial's argument rests on four articles. This is not, in itself, a disqualifying limitation, commentary pieces routinely work with small sets. The problem is that the text claims something stronger: that these four articles provide a “credible cross-section” of convergence as it “currently looks in the literature.” This is a representativeness claim that four self-selected papers cannot support.

The conflict-of-interest dimension warrants explicit acknowledgment: as editor of the Research Topic, Asgher (2025) selected the articles that are subsequently used as evidence for the thesis he advances. Standard epistemological hygiene requires that this circularity be named and mitigated, for instance through explicit acknowledgment of selection criteria, reference to the broader literature, or systematic comparison with excluded submissions. None of these mitigating moves are made.

The internal evidential quality is also uneven. The most prominently cited empirical study, Jiang et al.'s EEG investigation of LLM-assisted reasoning, reports results from 12 participants performing problem-solving tasks, with LLM interaction operationalized via GPT-4 in an unspecified chat-based interface. The primary datasets used (DEAP, AMIGOS, SEED, DREAMER) are affective computing benchmarks not designed to measure cognitive dynamics during LLM interaction, and their use for this purpose requires construct validity arguments that are not provided. Asgher (2025) himself notes the “construct-validity critiques” this invites, but then proceeds to characterize Jiang et al. as “the clearest BCI + LLM interaction bridge in the set,” a judgment that the methodological caveats do not support.

## 4 AI alignment: an open problem treated as engineerable

The editorial devotes particular attention to Edwards' contribution, which proposes an observer-centric, functional-contextual, neuro-symbolic approach to AI alignment. Edwards targets emergent Theory of Mind (ToM) as a computational capability rather than a philosophical label, and argues that alignment cannot be reduced to surface safety prompts but requires explicit representational machinery: values specification using ACT-inspired framing, utility estimation, and perspectival reasoning to guide LLM token selection. The editorial endorses this view, stating that Edwards' most valuable contribution is the insistence that alignment must be engineered as a visible layer of the system, anchoring the ethics and safety pillar in an explicit computational vocabulary. This includes structured interpretability via hypergraph representations of ToM-relevant relations.

The treatment of the Edwards contribution on AI alignment illustrates a pattern of conceptual foreclosure. Asgher (2025) endorses the view that alignment “must be engineered as a visible layer of the system, not treated as an emergent byproduct of scale,” and frames this as the delivery of an “explicit computational vocabulary” for what is called the ethics and safety pillar.

The problem of AI alignment, ensuring that an increasingly capable system acts in accordance with human values and intentions, is among the most contested open problems in computer science and philosophy of mind. The computational Theory of Mind (ToM) approach advocated by Edwards is one position in a large and unresolved debate. Presenting it as a practical engineering prerequisite for robust HMI, without engaging the substantial critical literature, is misleading. Russell (2019)'s argument about the difficulty of preference specification, the documented failures of RLHF-trained systems to generalize intentions rather than mimic them (Christiano et al., 2017), and the theoretical arguments about specification gaming (Krakovna et al., 2020) are directly relevant and unaddressed.

More broadly, the framing of alignment as a “designed system layer” sidesteps the question of whose values are encoded, by whom, verified by what process, and revisable under what conditions. These are not engineering questions: they are political and institutional ones, and their absence from a text concerned with deploying neuroadaptive systems in clinical and industrial settings is a substantive gap, not a disciplinary omission.

## 5 The near-absence of ethical and social analysis

In the editorial, the deployment layer of the proposed socio-technical control loop requires integration into clinical and industrial workflows with safety monitoring, auditability, and governance. Asgher (2025) briefly lists trust calibration, cognitive offloading, overreliance, and changes in attentional allocation as new variables introduced by LLM interaction layers. He also acknowledges that the decisive next phase requires evaluation protocols that jointly assess decision quality, cognitive workload, trust, overreliance risks, robustness to drift, and governance compliance.

The most consequential weakness of the editorial is the near-total absence of ethical and social analysis. The systems described, real-time measurement of workers' or patients' cognitive states, AI adaptation of behavior based on neurophysiological inference, continuous monitoring embedded in clinical and industrial cyber-physical systems, raise urgent questions of privacy, informed consent, power asymmetry, and potential coercive use. None of these are substantively addressed.

“Trust calibration,” “cognitive offloading,” and “overreliance” are mentioned as variables introduced by LLM interaction layers, but without analysis. The identity of the subject doing the calibrating, the differential vulnerability of specific populations to overreliance, the conditions under which trust in AI systems becomes epistemically irrational, these questions are not raised. The deployment of neuroadaptive monitoring in industrial workplaces, where the power differential between employer and employee makes “informed consent” structurally problematic, is not mentioned.

This omission is not incidental. The critical literature on technology in human contexts, from the work of Benjamin (2019) on race and technology to Crawford (2021)'s analysis of AI as infrastructure of power, has established that the sociotechnical consequences of deploying measurement and inference systems are not separable from their technical design. A research agenda that treats governance as a technical requirement (“implementable, auditable, and resilient mechanisms”) without engaging the political economy of who audits, who benefits, and who bears risk, is producing incomplete science by design.

The clinical context makes this especially acute. The da Vinci surgical robot system analyzed by Li et al. is a high-cost technology disproportionately deployed in high-income health systems. Presenting it as the paradigmatic example of Industry 4.0 in neuroscience implicitly frames the entire convergence agenda around a model of technological development that excludes the majority of the world's clinical contexts. Low-resource settings, community health infrastructure, and equitable access are not mentioned.

## 6 An unfalsifiable agenda

The editorial concludes that the four published articles show convergence is a substantive research direction rather than a slogan. Its forward-looking call identifies the critical gap that matters most for deployment: the field is still largely assembling components, while the decisive next phase requires what Asgher (2025) terms integration science, encompassing shared benchmarks and evaluation protocols for decision quality, cognitive workload, trust, overreliance risks, robustness to drift, and governance compliance within closed-loop systems operating in realistic cyber-physical contexts.

The editorial concludes by calling for “integration science,” “shared benchmarks,” and “evaluation protocols that jointly assess decision quality, cognitive workload, trust, overreliance risks, robustness to drift, and governance compliance within closed-loop systems operating in realistic cyber-physical contexts.” This is a reasonable list of desiderata. It is not, however, a falsifiable research agenda.

No indicators of progress are specified. No criteria are provided by which a claim that the convergence is occurring, or has been achieved, could be evaluated. No benchmarks are proposed, despite the call for benchmarking. The result is what Smaldino and McElreath (2016) have called a “theoretical placebo”: a formulation sufficiently vague that any future development can be retrospectively interpreted as confirming it, and no development can conclusively disconfirm it.

A research agenda that merits the term should specify, at minimum: what measurements would indicate that the integration is succeeding, what findings would indicate that the convergence framework is wrong, and what the timeline and criteria for reassessment are. The editorial provides none of these.

## 7 The inferential gap between EEG signals and mental states

The editorial's measurement layer relies heavily on EEG-based neuroergonomics evidence from Jiang et al., whose pilot study with 12 participants reports three main findings: reduced frontal theta power (interpreted as lower cognitive workload), increased P300 amplitude (interpreted as enhanced attentional and decision integration), and lower NASA-TLX subjective workload ratings under LLM-assisted conditions. The editorial frames these as precisely the type of measurable neuroergonomics signals the Research Topic called for: evidence that LLMs can shift the cognitive economics of reasoning under defined interaction conditions. Moreover, the editorial suggests these quantifiable hooks (theta, P300, subjective workload) could be operationalized in future Industry 4.0 deployments as constraints or triggers for assistance adaptation, throttling, or escalation.

We reserve what we consider the most fundamental concern for last, because it cuts beneath the methodological and rhetorical issues to the epistemic foundation of the convergence project itself.

The entire logic of the four-layer loop, measure human cognitive state, infer, adapt, deploy, depends on the assumption that EEG and related physiological signals provide reliable access to the mental states they are claimed to index. This assumption is less secure than the editorial implies, in ways that matter for the systems being designed.

EEG measures fluctuations in the electromagnetic field at the scalp surface, the aggregate electrical consequence of millions of synchronizing neurons. It does not measure attention, cognitive load, or affect as theoretical constructs. It measures a downstream physical correlate of processes occurring at multiple levels of neural organization that remain incompletely understood. When Jiang et al. report that frontal theta power decreases under LLM-assisted conditions and interpret this as evidence of “reduced cognitive load,” they are making an inferential move that traverses several layers of theoretical assumption: from raw signal to frequency band power, from frequency band power to cognitive construct, from cognitive construct to phenomenological state. Each transition involves acknowledged uncertainties that are rarely propagated through to the final interpretation.

This is not a criticism unique to Jiang et al., it applies broadly to applied EEG research. But the scale of the inferential leap matters when the downstream application is a neuroadaptive system that adjusts its behavior based on inferred mental states, and when the claim is that such systems can “measure human cognitive and affective states” in real operational environments. The gap between measuring an electromagnetic correlate and knowing a mental state is precisely the gap that the hard problem of consciousness (Chalmers, 1995) marks as unresolved: we do not know whether phenomenal experience is identical to, realized by, or emergent from physical processes, and the answer matters for what we are licensed to claim when we say a system has “measured” a cognitive state.

Applied neuroscience routinely operates with a methodological correlationalism that brackets this question, and for many predictive purposes, this is scientifically defensible. The problem arises when the bracketing is forgotten, and correlates are described as if they were direct measurements of the states they correlate with. The editorial consistently makes this move, describing the measurement layer as providing access to “human cognitive and affective states” rather than to electrophysiological signals that covary with behavioral and subjective reports under specific task conditions.

The consequence for the convergence agenda is significant. A closed-loop neuroadaptive system that adapts AI behavior based on inferred mental states is not adapting to what a person is experiencing: it is adapting to a model of what a person is experiencing, built on correlates whose relationship to experience is theoretically underspecified. This is not a reason to abandon the project, but it is a reason to be considerably more cautious about what such systems can and cannot do, and about the risks of deploying them in high-stakes contexts where the cost of a miscalibrated inference is borne by a patient or worker who cannot easily contest it.

“*The question is not whether EEG correlates of cognitive states exist, they do, but whether those correlates are sufficient to license the inferential and operational moves that the convergence architecture requires. The editorial does not engage this question. It should.”*

## 8 Acknowledgment of genuine contributions

Intellectual honesty requires acknowledging what the editorial does well. The four-layer conceptual model, interaction, measurement, inference, deployment, is a useful heuristic that helps position contributions from disparate sub-fields within a common architecture. The insistence that LLMs be treated not only as tools but as cognitive variables that modify human reasoning, and that this modification be measured neurophysiologically, is a methodologically mature position that goes beyond most of the applied AI literature. The frank acknowledgment that the field is still assembling components, even if inconsistently framed, signals a sober realism that contrasts favorably with more triumphalist pronouncements about AI readiness.

These contributions are real. The problem is that the editorial's ambitions exceed its foundations. An orientating document for a fragmented community has different standards than a foundational paper claiming to define an empirically grounded convergence. Asgher (2025)'s text is closer to the former but presents itself as the latter.

## 9 Conclusion

Asgher (2025)'s editorial is a useful signal of direction. It is not, by the standards it implicitly invokes, a rigorous foundation for the agenda it proposes. Its principal weaknesses, the assertion rather than demonstration of convergence, the insufficient empirical base, the oversimplification of alignment, the near-total absence of ethical analysis, the unfalsifiable agenda, the acritical use of Industry 4.0, and most fundamentally the unexamined inferential gap between EEG signals and mental states, are structural, not peripheral.

We close with a methodological proposal. A genuinely convergent research program for neuroadaptive human–AI systems should explicitly specify: (a) the inferential chain from signal to mental state, with uncertainty estimates at each step; (b) the ethical framework governing deployment, including consent, audit, and contestability mechanisms; (c) falsifiable milestones by which progress toward integration can be assessed; and (d) explicit engagement with what it would mean for the convergence thesis to be wrong. Absent these elements, the field risks mistaking a persuasive narrative for a scientific program, a substitution that is consequential precisely because the systems being designed will act on real people in real environments.
